# Calcium phosphate mineralization is widely applied in crustacean mandibles

**DOI:** 10.1038/srep22118

**Published:** 2016-02-24

**Authors:** Shmuel Bentov, Eliahu D. Aflalo, Jenny Tynyakov, Lilah Glazer, Amir Sagi

**Affiliations:** 1 Dept. of Life Sciences and the National Institute for Biotechnology in the Negev, Ben-Gurion University, Beer Sheva, Israel; 2 Department of Biology, Woods Hole Oceanographic Institution 39 Water St., Woods Hole, 02543, MA, USA

## Abstract

Crustaceans, like most mineralized invertebrates, adopted calcium carbonate mineralization for bulk skeleton reinforcement. Here, we show that a major part of the crustacean class *Malacostraca* (which includes lobsters, crayfishes, prawns and shrimps) shifted toward the formation of calcium phosphate as the main mineral at specified locations of the mandibular teeth. In these structures, calcium phosphate is not merely co-precipitated with the bulk calcium carbonate but rather creates specialized structures in which a layer of calcium phosphate, frequently in the form of crystalline fluorapatite, is mounted over a calcareous “jaw”. From a functional perspective, the co-existence of carbonate and phosphate mineralization demonstrates a biomineralization system that provides a versatile route to control the physico-chemical properties of skeletal elements. This system enables the deposition of amorphous calcium carbonate, amorphous calcium phosphate, calcite and apatite at various skeletal locations, as well as combinations of these minerals, to form graded composites materials. This study demonstrates the widespread occurrence of the dual mineralization strategy in the *Malacostraca*, suggesting that in terms of evolution, this feature of phosphatic teeth did not evolve independently in the different groups but rather represents an early common trait.

Biomineralization in the major animal phyla began during the Cambrian explosion that occurred approximately 525 Myr ago. The minerals used by most animals for skeletal reinforcement were calcium salts; either calcium phosphate (CaP) or calcium carbonate. As a result, these two minerals became the most important bioinorganic materials throughout the Phanerozoic period until present times, both in terms of phylogenetic distribution and in terms of biomineral quantities^1^.

After a poorly delineated period during which both calcium phosphate and calcium carbonate were used by organisms of uncertain phylogenetic affinities (“Problematica”), the major phyla that subsequently ensued specialized in formation of either CaP or calcium carbonate. According to the currently held view of biomineral distribution, vertebrates adopted the use of calcium phosphate for their endoskeleton while invertebrates use calcium carbonate for their exoskeleton^2^. (The two known exceptions are the inarticulate brachiopods *Lingulata* and the *Iblidae* barnacles, each of which use CaP^3^,^4^). Specialization of the major taxa according to the “preferred” principal biomineral is thought to have been established during the Ordovician period (488–444 Myr ago) and became a highly conservative feature which still characterizes extant phyla^5^.

Arthropods, like other invertebrates, rely on calcium carbonate as their principal reinforcing mineral. Still, reports of crustaceans containing variable amounts of phosphorous within their carbonate skeletons are known^1^,^6^,^7^,^8^,^9^,^10^,^11^. Indeed, this phenomenon was observed by Darwin who noted that crustacean calcareous shells show a much higher variability in terms of phosphate content as compared with the molluscan shell, which have virtually no phosphate^12^.

The presence of phosphate in crustacean skeletons was mainly attributed to co-precipitation with amorphous calcium carbonate (ACC)^13^,^14^, with a supposed function of ACC stabilization^9^,^15^. The presence of apatite crystals in skeletal tissue is considered most unusual and was thought to be restricted to the barnacle group of *Iblidae* that contain small crystals of carbonated apatite^4^. However, Bentov *et al.*^16^ recently showed that the molar teeth of the crayfish *Cherax quadricarinatus* are reinforced by large oriented crystals of calcium phosphate in the form of fluorapatite. The phosphatic cover in these molars is mounted over a carbonate basis, forming a graded structure of multi-phase composite material that results in supreme mechanical properties comparable to vertebrate enamel. A similar instance of large, oriented, apatite crystal was reported for the raptorial appendage of a mantis shrimp^17^,^18^.

In view of the general tendency of crustaceans to incorporate phosphate into their calcareous skeletons and the recent reports of elaborate apatite structures in crustaceans^16^,^17^, it seems that a re-evaluation of phosphate precipitation in crustaceans is warranted. Teeth seem to be appropriate skeletal elements in which to study calcium phosphate reinforcement, as they have evolved biomechanically to withstand the massive compressional bending and torsional stresses associated with feeding^19^. Indeed, in many organisms, teeth represent the hardest part of the body, presenting superior resistance and biomechanical qualities. In invertebrates, some of the fascinating mechanisms for material strengthening are found in the feeding tools^20^,^21^,^22^,^23^,^24^. Calcium phosphate offers a clear advantage in the strengthening of feeding tools. In its crystallized forms, apatite represents the hardest option in the range of calcium carbonate/phosphate salts. Also in the amorphous phase, it was found that an increased amorphous calcium phosphate (ACP)/ACC ratio is associated with increased mechanical qualities, such as hardness and elastic modulus^16^,^25^. In addition, the solubility product of ACP^26^ presents a higher acid resistance than do carbonates, even when compared with the most stable CaCO_3_ polymorph, calcite.

In the present study we question whether the documented deposition of crystalline calcium phosphate in teeth of the mandibles of C. *quadricarinatus* is a rare phenomenon or, rather, represents a wider pattern in crustaceans. To answer this question, we compare the mineralogy and chemistry of the teeth processes in seven different taxa of crustaceans to the “basal segment”, which is analogous to the crustacean “jaw” and is similar to the bulk cuticle.

## Results

**Figure 1:**
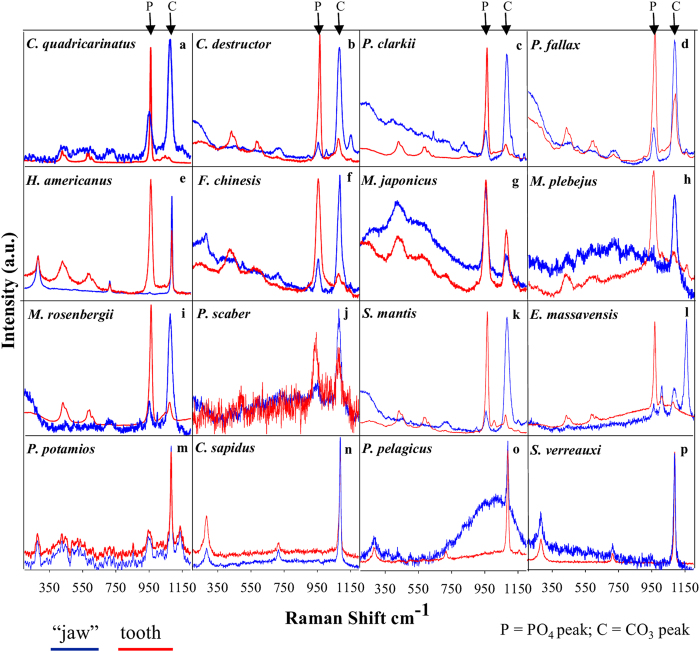
Raman spectra of teeth processes (red) and the underlying mandibular basal segment (jaw; blue) of 16 representative crustacean species from the Class Malacostraca; Astacidea (**a–e**), Dendrobranchiata (**f–h**), Caridea (**i**), Isopoda (**j**), Stomatopoda (**k,l**) Brachyura (**m–o**), Achelata (**p**). The Raman peak at 960 cm^−1^ corresponds to calcium phosphate (ν^1^ vibration of PO_4_) and the peak at 1080 cm^−1^ corresponds to calcium carbonate (ν^1^ vibration of CO_3_). The list of species studied is presented in Table 1.

**Figure 2:**
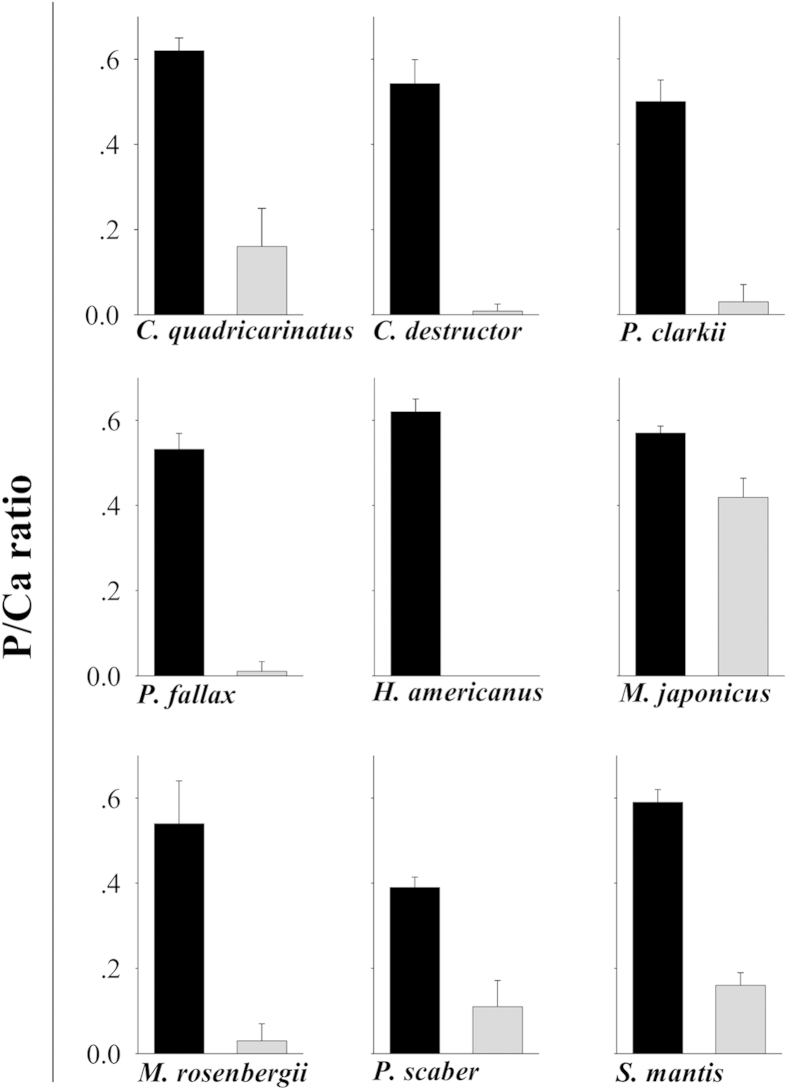
EDS analysis comparing P/Ca ratios in the teeth processes (black) and the mandibular basal segment (gray) of 9 representative crustacean species (see Table 1) that deposit calcium phosphate on their teeth. Error bars are s.d. for the typical atomic ratio of various calcium phosphate minerals (see Fig. S2).

**Figure 3:**
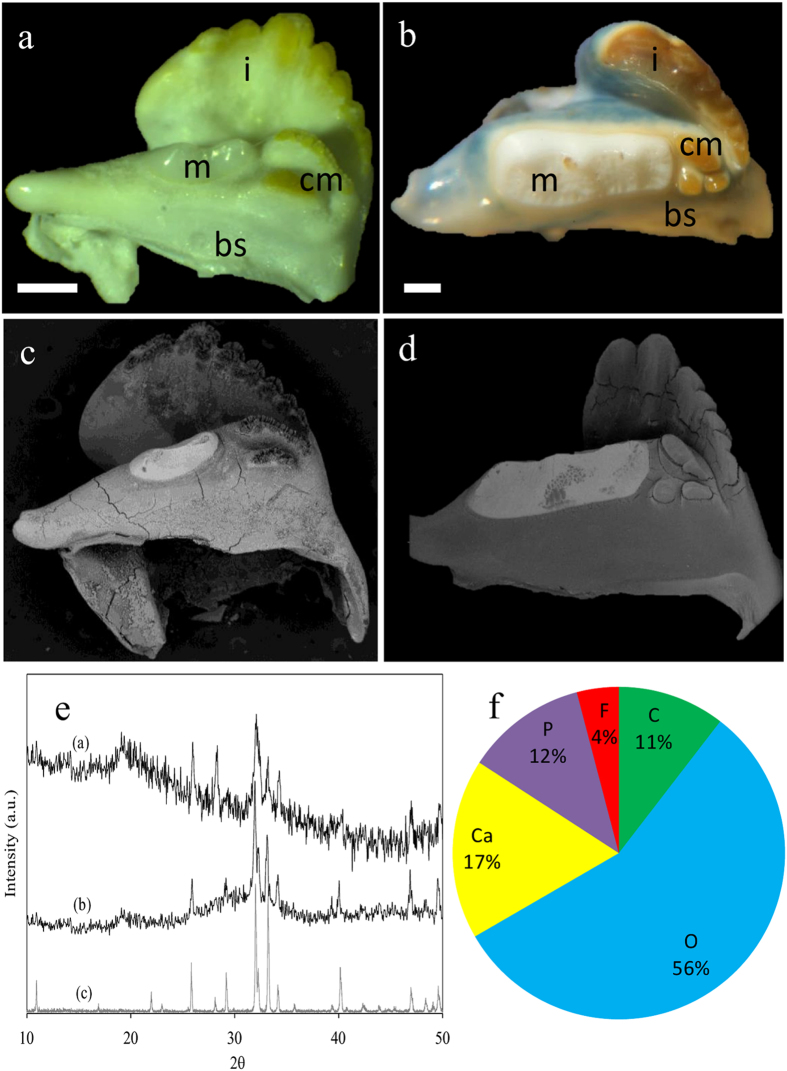
The mandibles of the crayfish *C. quadricarinatus*, and *P. fallax (Astacidea)*. (**a**) Micrographs of the mandible of *P.fallax* demonstrate the porcelaneous rounded molar cusp (m) composed of apatite, and the bicuspid caudal molar (cm) and the incisor tooth (i), which are covered with chitin and are reinforced with ACC. (**b**) The mandible of *C. quadricarinatus* with anterior whitish molar tooth, and brownish tricuspid caudal molar process and incisor ridge that are covered by chitin (i-incisor, m-molar, bs-basal segment, scale bar = 1 mm). (**c,d**) the correspondent backscattered images showing the sharp gradient of mineral density between the anterior phosphatic molar and the rest of the mandible. (**e**) X-ray diffraction of the molar teeth of, *P. fallax* (**a**) and *C. quadricarinatus* (**b**). The diffractogram demonstrates that the molar tooth in these species is comprised of crystalline apatite. The diffraction peak at 2θ = 19.2 is ascribed to the associated chitin matrix (lattice plane (040), (110)). **(f)** EPMA-WDS analysis of *C. quadricarinatus* molar tooth indicates on a composition of fluorapatite. Part of the carbon and oxygen signal is probably due to the organic matrix, yet it is possible that part of the carbon signal is due to carbonate-fluorapatite (francolite).

**Figure 4:**
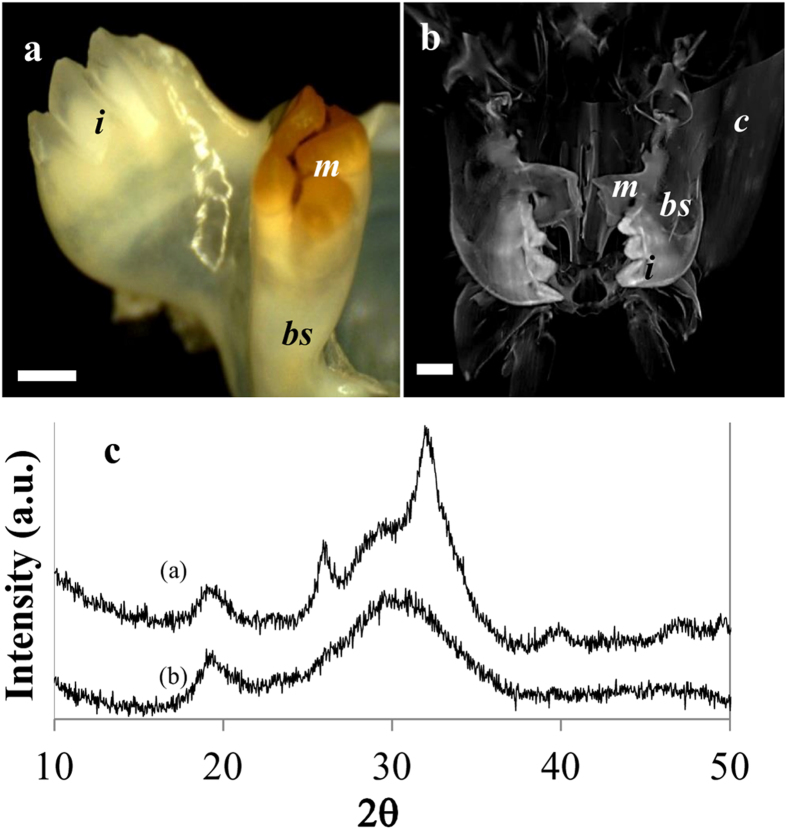
Mandible of the prawn, *M. rosenbergii*. (**a**) Micrographs of the teeth processes illustrate the reverse distribution of chitin coat in the mandible compared to crayfish. In *M. rosenbergii,* the incisors are highly mineralized while the molar is less mineralized and is coated with chitin. (**b**) X-ray computer tomography of the mouth parts area demonstrates the gradual changes of mineral density from the teeth processes to the jaw and the rest of the head. The bulk cuticle is relatively lightly calcified with ACC while the mandibles show high mineral density, especially in the incisors (i-incisor, m-molar, bs-basal segment, c-cuticle, scale bar = 2 mm). (**c**) Powder XRD pattern of the molar (**a**) and the incisor (**b**) teeth. The diffractogram indicates that the molar tooth is composed of apatite while the incisor tooth is composed of ACP.

**Figure 5:**
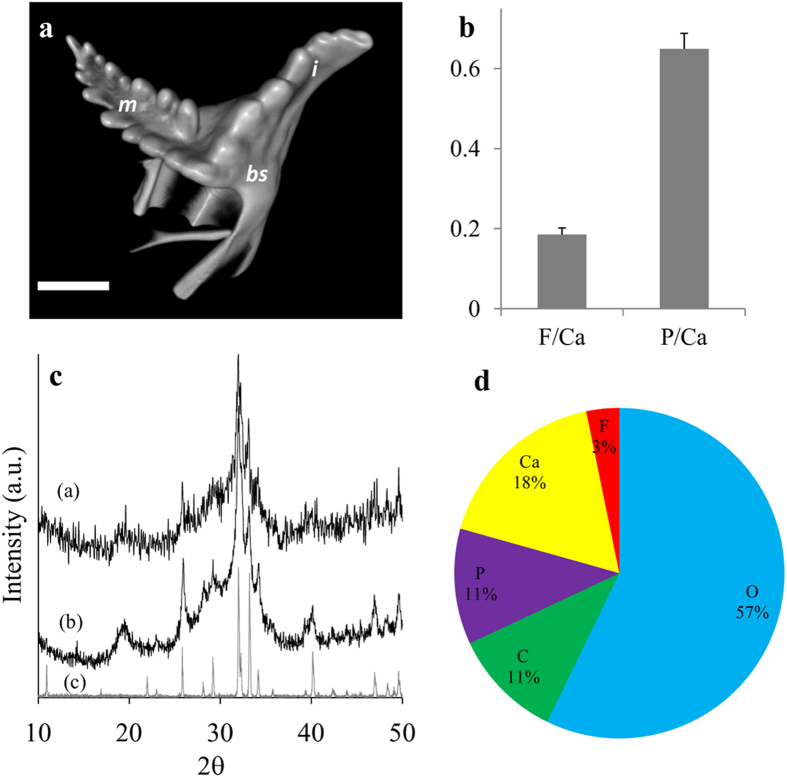
Mandible of mantis shrimps *S. mantis*, and *E. massavensis*. (**a**) Micro-CT volume rendering reconstructions of a surface view of the *S. mantis* mandible showing two serrated ridges of the molar and a single serrated ridge of the incisor. (i-incisor, m-molar, bs-basal segment, scale bar = 2 mm). A volume-rendered video of the mandible is available as Supplementary Video S1. (**b**) EDS analysis of the teeth of *E. massavensis* showing ratios of F/Ca and P/Ca, confirms a chemical composition of fluorapatite (Ca_5_(PO_4_)_3_F). Error bars are s.d. (**c**) Powder X-ray diffraction of the mandibles, *S. mantis* (**a**), *E. massavensis* (**b**) fluorapatite reference (**c**), demonstrates that the teeth are comprised of crystalline apatite (for peak assignment see Fig. 3). (**d**) EPMA-WDS analyses of *S. mantis* teeth show a composition of fluorapatite, possibly carbonate-fluorapatite (francolite).

The chemical and mineral compositions of mandibles from seven different taxa (sixteen species) of crustaceans were examined. We compared the compositions of the external chewing/cutting surfaces of the teeth, tissues that would probably benefit from phosphatic extra hardening, to the proximal basal segment that is comparable to the jaw and is similar to the bulk of the exocuticle. The analyses were concentrated at the mineralized outer surface of teeth/jaw, thus, representing the mineralogy of the exocuticle. Mandible mineralogy was determined by Raman spectroscopy (Fig. 1), whereas the corresponding chemical compositions were assessed by EDS (energy dispersive spectrometry) (Fig. 2). Five species that showed Raman spectra with a clear pattern of crystalline apatite were analyzed by powder X-ray diffraction (Figs 3, 4, 5).

The Raman spectra (Fig. 1) reveal the mineralogy of the teeth processes (red) and the jaw (“basal segment”, blue) of sixteen crustaceans from the Astacidea (A-E), Dendrobranchiata (F-H), Caridea (I), Isopoda (J), Stomatopoda (K-L), Brachyura (M-O) and Achelata (P). Raman bands at ~960 cm^−1^ (reddish shade) are attributed to calcium phosphate (υ_1_ mode of PO_4_) in the form of ACP (<960 cm^−1^) or apatite (>960 cm^−1^), while bands at ~1080 cm^−1^ (bluish shade) are attributed to calcium carbonate (υ_1_ mode of CO_3_) in the form of ACC (<1083 cm^−1^) or calcite (>1083 cm^−1^). At lightly calcified areas of the jaw, as in *E. massavensis* (Fig. 1l), the spectra are dominated by the carotenoid pigment astaxanthin with a typical peak at ~1150 cm^−1^ (υ_2_ of C-C single bond stretching mode). Standard control Raman spectra of hydroxyapatite, fluorapatite, ACP, calcite and ACC are shown in Supplementary Fig S1.

EDS-based chemical analysis of mandibles that contain calcium phosphate from several species (Fig. 2) demonstrates the different P/Ca ratios of the teeth (molars or incisors), as compared to the basal segment. The P/Ca atomic ratios of the teeth are compatible with those of apatite or ACP, while the lower P/Ca ratio of the jaw is typical of ACC enriched with phosphate. For typical P/Ca ratios of related calcium phosphate minerals see Supplementary Fig. S2.

The results show that the formation of calcium phosphate teeth at the mandibles, takes place in Astacidea (crayfish and lobsters), Caridea (prawns), Dendrobranchiata (shrimps), Isopoda and Stomatopoda (Mantis shrimps) (Fig. 1a–l). According to the Raman spectra, EDS and XRD analyses, the mineralogy of the calcium phosphate in these species is in the form of either apatite or ACP. Crystalline apatite was identified in the mandibles of five species (Figs 3, 4, 5) while other phosphatic species show ACP or poorly crystalline apatite. In the representatives of two infraorders, Brachyura and Achelata (crabs and spiny lobsters, respectively), deposition of calcium phosphate at the feeding tools was not detected, with only crystalline calcite being found in their mandibles (Fig. 1m–p). Interestingly, it seems that in these species, unlike the others, ACC is absent from the underlying jaw as well.

The distribution of calcium phosphate within the crustacean mandible is not uniform. In some species, calcium phosphate is found in the molars, in other species it is found in both structures; molars and incisors. Of the species examined, *C. quadricarinatus, S. mantis, E. massavensis, P. fallax and M. rosenbergii* show the most elaborate apatite crystals. In the crayfish mandible, the anterior molar tooth, usually with a porcelaneous appearance (Fig. 3a,b), is composed of CaP while the caudal molar and the incisor comprise CaCO_3_ frequently covered with chitin. Backscattered images of the mandibles (Fig. 3c,d) demonstrate the high density of the phosphatic molar tooth compare with the rest of the mandible. XRD analyses (Fig. 3e) confirmed the crystallinity of the molar tooth and showed the typical diffraction peaks of apatite. All samples show a diffraction peak at 2θ = 19° which corresponds to lattice planes (110) of chitin. EDS analysis of the molar tooth of the crayfish *P. fallax* show a F/Ca ratio of 0.21 (±0.06). EPMA-WDS analysis of the molar tooth of *C. quadricarinatus* (Fig. 3f, Supplementary Table S1) indicates a composition of fluorapatite, possibly carbonate-fluorapatite.

In the Caridea representative, *M*. *rosenbergii*, both molar and incisor teeth are composed of calcium phosphate. The mineral density of the different teeth seems to have an opposite pattern of that of the crayfish. In the Caridea, the incisors are heavily mineralized while the molars are less mineralized and are covered by a brown chitinous layer (Fig. 4a). X-ray computer tomography of the mandible of *M. rosenbergii* (Fig. 4b) demonstrates the sharp mineral density contrast between the teeth processes (especially the incisors) and the rest of the cephalon (head), which is lightly calcified with ACC. The density gradients at the incisors probably indicate the existence of a mechanically graded structure, as was shown for the molar of *C. quadricarinatus*^16^. Interestingly, in this species the crystallinity degree does not seem to correlate with the mineral density. Powder XRD (Fig. 4c) clearly demonstrates that the molars contain crystalized apatite while the incisors seem to be composed exclusively of ACP.

In the mantis shrimps (Stomatopoda) *S. mantis* and *E. massavensis* (Fig. 5), the molars and the incisors are heavily mineralized with crystalline apatite. The molar is composed of two saw-like ridges that are similar to the single ridge of the incisor (Fig. 5a, supplementary video S1). Powder XRD analysis (Fig. 5c) showed that the teeth are composed of crystalline apatite. EDS analysis of *E. massavensis* teeth (Fig. 5b) and WDS analysis of *S. mantis* teeth (Fig. 5d, Supplementary Table S2), shows that similar to the situation in Astacidea, the apatite have a composition of fluorapatite, possibly carbonate-fluorapatite.

## Discussion

The present study suggests that calcium phosphate (CaP) biomineralization is widely employed in crustacean teeth. In general, calcium carbonate is used for the bulk reinforcement of the cuticle while calcium phosphate is used selectively for mechanically challenged sites. In light of the present study, and previous studies^16^,^17^,^25^, it seems that phosphate is not merely a co-precipitate of the calcium carbonate system but rather that calcium phosphate is deposited in a controllable mode that probably serves specific mechanical and chemical functions. The CaP/CaCO_3_ ratio seems to be finely tuned by a regulatory system able to orchestrate the two biomineralization processes so as to produce various combinations of the two minerals. The use of each calcification system probably requires a different ion regime, different proteinaceous machineries, etc.^27^,^28^, implying that many crustaceans possess a biomineralization “toolkit” for calcium phosphate formation as well as for calcium carbonate formation.

Phosphate mineralization can contribute in both the amorphous and crystalline phases. In its crystallized form, apatite offers the hardest mineral option from the range of calcium carbonate/phosphate salts (on the Mohs hardness scale, apatite is 5 while calcite is 2.5–3^29^). Likewise, in the amorphous phase, calcium phosphate probably plays a major role in enhancing the mechanical properties and chemical resistance of the exoskeleton. Usually, ACP is associated with ACC in a homogeneous mixture of solid solution^30^, where it probably plays a role in the stabilization of ACC^9^. Mechanical analysis of the crayfish mandible^16^ and the mantis shrimp dactyl limb^25^ shows that there is a positive correlation between ACP/ACC ratio to hardness and elasticity. In addition, ACP probably contributes to chemical resistance of the skeletal element due to its low solubility, as compared to ACC and even to calcite^26^. One of the abundant natural solutions for reinforcement of a skeletal structure with the aim of withstanding massive impact, is the formation of a mechanically graded structure, in which hard material is mounted over a softer base that absorbs the impact and deflects potentially dangerous cracks^31^. It seems that the dual mineral system described in the present study provides the animal with an efficient method of forming mechanically graded structures through a gradual change in the phosphate/carbonate ratio, as demonstrated here in the mandibular structure of crustaceans.

Regarding the crystalline phase, our results (Figs 3f and 5b,d, and supplemental Tables S1, S2), and previous data^16^, suggest that the crystalline calcium phosphate that appears in crustaceans is frequently in the form of fluorapatite. The fact that no traces of fluoride were found in the ACP phases suggests that fluoridation is regulated and restricted to the crystalline phase. The formation of fluorapatite rather than hydroxyapatite might serve some functional role, such as enhanced crystal growth rate, increased crystallinity and reduced solubility^32^,^33^.

Unlike representative crustaceans of other groups, the Brachyura and Achelata species investigated here did not reveal calcium phosphate enrichment in their teeth processes. Interestingly, specimens from both groups were also the only ones that did not show any ACC in their basal segments. The reason for the absence of calcium phosphate in the mandibles of Brachyura and Achelata is not known. However, it might be associated with the general shift of these species to a heavy armor skeleton (which may also be associated with their loss of escape-related giant neurons^34^). The need for phosphatic reinforcement of the mandible is more critical in crustaceans with an exoskeleton that is based on soft and soluble ACC as compared with robust crustaceans. It is also possible that the mechanical advantage of an apatite crown is apparent only when mounted over a softer amorphous sub-layer. Mounting a hard and brittle calcium phosphate layer over hard calcite may result in a fragile tooth.

### The possible connection between feeding strategy and CaP distribution patterns in the incisor and molar

The distribution of CaP within the mandible differs in different taxa. In the *Astacidea*, molars are composed of CaP while incisors are reinforced with calcium carbonate (Fig. 3). In the *Caridea* both molar and incisor processes are reinforced with CaP, however, the incisors show a higher mineral density than the molars (Fig. 4). In Stomatopoda, both incisors and molars are reinforced by crystalline fluorapatite (Fig. 5). It is plausible that these different distribution patterns reflect different habitats and feeding behaviors. It can be hypothesized that CaP incisors probably reflect the need for enhanced grasping tearing and biting force while CaP molars reflect feeding habits that require enhanced mastication and grinding efficiency. It is noteworthy that although the initial handling of food and prey is usually performed by the claws (chelipeds), however, due to higher mechanical leverage of the mandibular adductor, the mechanical advantage (MA) of the mandible bite is much higher than that of the claws^35^. The heavy mineralization of the mandible probably evolved correspondingly to withstand the potential high mechanical stress during biting and/or mastication. Calcium phosphate teeth projections, with improved hardness and wear resistance, are probably located where the mechanical load is higher.

### Dual mineralization system in malacostracan crustaceans: an evolutional perspective

It was previously reported that the mandible of the crayfish *C. quadricarinatus* has an “enamel-like” apatite cover on the molar tooth, representing a unique case of convergent evolution with vertebrates in which a similar solution has developed for similar functional challenges^16^. In this paper we show that this convergent evolution is not a unique case but rather represents a common trend in the Malacostraca. The bulk exoskeleton is reinforced with CaCO_3_ while the teeth processes are frequently composed of calcium phosphate as a major constituent. The wide occurrence of CaP reinforcement in the mandibles raises the possibility that the calcium phosphate mineralization mechanism did not evolve independently in the different groups but rather has older evolutionary roots. The Malacostraca, the largest class of the Crustacea, is one of the two major lineages of crustaceans (along with the “Entomostraca”), with a fossil record that goes back to the Cambrian^36^. The divergence of the stomtopod group (Hoplocaridae) from other malacostracans is very early, with fossil evidence indicating that they diverged in the Devonian^37^. In agreement, phylogenetic analyses based on hemocyanin subunits suggest a divergence time of ~405 MYA^38^. Thus, the similar fluorapatite teeth of the Stomatopoda (Hoplocarida) and Eumalacostraca may support the assumption that the dual mineral system in both groups has common evolutionary roots.

In exploring the morphology of early arthropods for innovations that led to basic arthropod design and body plan, various novelties have been identified^39^,^40^,^41^. These include the evolution of sclerotization, external segmentation, and jointed processes. It is widely assumed that one of the major driving forces for these rapid changes is an “arms race” for improved predation and protection^42^. Among the arthropods, the “arms race” of the Cambrian explosion may well have been a “limbs race”^43^ in which the mouth parts and specifically the mandibles developed and became a characteristic morphological feature of the stem group Mandibulata (crustaceans, insects and myriapods). Mandible and cuticle sclerotization by mineral-reinforcement also probably evolved as part of this “arms race”. A hardened cuticle entails many other changes in the bodies and life cycles of these early arthropods; growth required a sequence of cuticular molts, and locomotion required arthropodization. Hardening by mineralization raises inherent conflicting considerations, such as durability versus flexibility. Moreover, periodic molting gives extra importance to the metabolic price paid for precipitation of each mineral phase and its resorbability potential. It is possible that one solution that evolved for these conflicts was the recruitment of a dual mineral system for the formation of calcium carbonate and calcium phosphate. Such a dual system offers the obvious advantage of versatility, enabling the formation of various minerals and their combination at different body locations to form graded composite structures. It may also enable a fast response (on an evolutionary time scale) to various selective pressures, including the secular variations in ocean chemistry such as the phosphogenic events of the early Cambrian^44^ and the Permian–Triassic catastrophic carbon dioxide increase^2^ that dramatically changed the applicability of calcium carbonate and calcium phosphate. Therefore, a dual mineral system might be considered as one of the factors that contributed to the survival and success of malacostraca crustaceans.

## Methods

### Animals

For this study, representative species of the clades *Astacidea, Caridea, Dendrobranchiata, Brachyura, Achelata, Isopoda* and *Stomatopoda* were collected. The species selected are listed in Table 1.

Raman spectroscopy: Raman spectra were acquired on a Jobin–Yvon LabRam HR 800 micro-Raman system equipped with a liquid nitrogen-cooled detector. Excitation was achieved with a He–Ne laser line (633 nm). A X50 microscope objective, a 100-μm confocal hole, and a 600 grooves/mm grating were used, giving a resolution of 2–4 cm^−1^. The different spectra represent the Raman scattering from the outer mineralized surfaces of the teeth and the basal segment (“jaw”).

SEM and energy dispersive spectrometry (EDS) analysis: Dried samples were sputter-coated with platinum or gold and examined using a JEOL JSM-7400f apparatus. Error bars represent standard deviation. It is noteworthy that in backscattered images of the mandibles, the intensity of the backscattered electrons (gray level) is the result of both bulk mineral density (mineral/organic ratio) and the (mean) atomic number of the mineral. Different minerals have a different mean atomic number. e.g. calcite and apatite (12.565 and 14.068 respectively^45^), that consequently display a different backscattering coefficient.)

Wavelength-Dispersive X-Ray Spectroscopy (WDS) analyses: The teeth were analyzed with a JEOL 8230 Superprobe EPMA with four wavelength-dispersive spectrometers for microanalysis (at the Institute of Earth Sciences, Hebrew University). Beam conditions were set to 15 keV and 15 nA. All phases were analyzed for all major elements; Ca, P, F, O, and C. The system was calibrated using analytical standards AS 02753-AB 53 Minerals Standard from SPI for different elements. Data was processed using a ZAP correction procedure.

Powder X-ray diffraction: Analysis was performed on milled samples of external layers of the teeth spread on a quartz plate and a Philips model PW-1050/70 diffractometer.

Micro-CT: Scans were performed on a Skyscan1 1172 micro-CT machine (Skyscan). The X-ray source was set at 60 keV and 160 mA, with a resolution of 3.5 μm pixels. Specimens were scanned through 360**°** with 0.7**°** rotation increments. The projection images were reconstructed using the NRecon software (Skyscan). Volume renderings reconstructions were performed using CTVox software (Skyscan). In Supplementary Video S1, gray-scale images were pseudo-colored according to an RGB palette, with low mineral density in red and high density in blue.

## Additional Information

**How to cite this article**: Bentov, S. *et al.* Calcium phosphate mineralization is widely applied in crustacean mandibles. *Sci. Rep.* **6**, 22118; doi: 10.1038/srep22118 (2016).

## Supplementary Material

Supplementary Information

Supplementary Video S1

## Figures and Tables

**Table 1:** List of species addressed in this study.

Taxon	Species	Common name
*Astacidea*	*Cherax quadricarinatus*	Australian red claw crayfish
	*Cherax destructor*	Common yabby
	*Procambarus clarkii*	Red swamp crayfish
	*Procambarus fallax*	Slough crayfish
	*Homarus americanus*	American lobster
*Caridea*	*Macrobrachium rosenbergii*	Giant river prawn
*Dendrobranchiata*	*Marsupenaeus japonicus*	Japanese tiger shrimp
	*Melicertus plebejus*	Eastern king prawn
	*Fenneropenaeus chinensis*	Chinese white shrimp
*Isopoda*	*Porcellio scaber*	Common rough woodlouse
*Achelata*	*Sagmariasus verreauxi*	Eastern rock lobster
*Brachyura*	*Potamon potamios*	Aquatic land crab
	*Callinectes sapidus*	Atlantic blue crab
	*Portunus pelagicus*	Flower crab
*Stomatopoda*	*Squilla mantis*	Mediterranean mantis shrimp
	*Erugosquilla massavensis*	Indo-Pacific mantis shrimp
