# Supplementary material for: Calcium phosphate mineralization is widely applied in crustacean mandibles
Source: Sci Rep. 2016 Feb 24;6:22118. doi: 10.1038/srep22118 (PMC4764981; doi:10.1038/srep22118)
Supplement: Supplementary Information [file srep22118-s1.pdf]

Calcium phosphate mineralization is widely applied in crustacean mandibles.

Authors: Shmuel Bentov*, Eliahu D. Aflalo , Jenny Tynyakov Samra , Lilah Glazer and Amir Sagi*

Supplementary Information

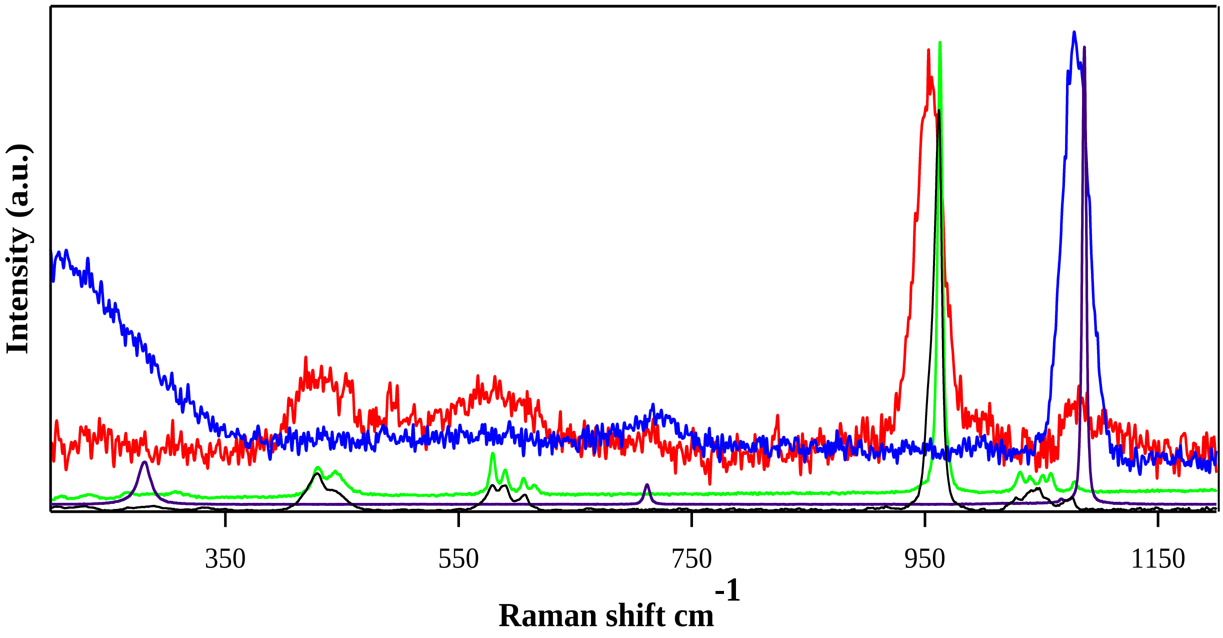

Figure S1. Raman spectra of fluorapatite (green), hydroxyapatite (black), ACP (red), calcite (dark blue) and ACC (blue). The Raman peak at 960 cm^{-1} corresponds to calcium phosphate (ν^1 vibration of PO_4) and the peak at 1085 cm^{-1} corresponds to calcium carbonate (ν^1 vibration of CO_3). The amorphous phases, ACC and ACP, show a typical shift to a lower wave number and a peak broadening (FWHM >25 cm^{-1}).

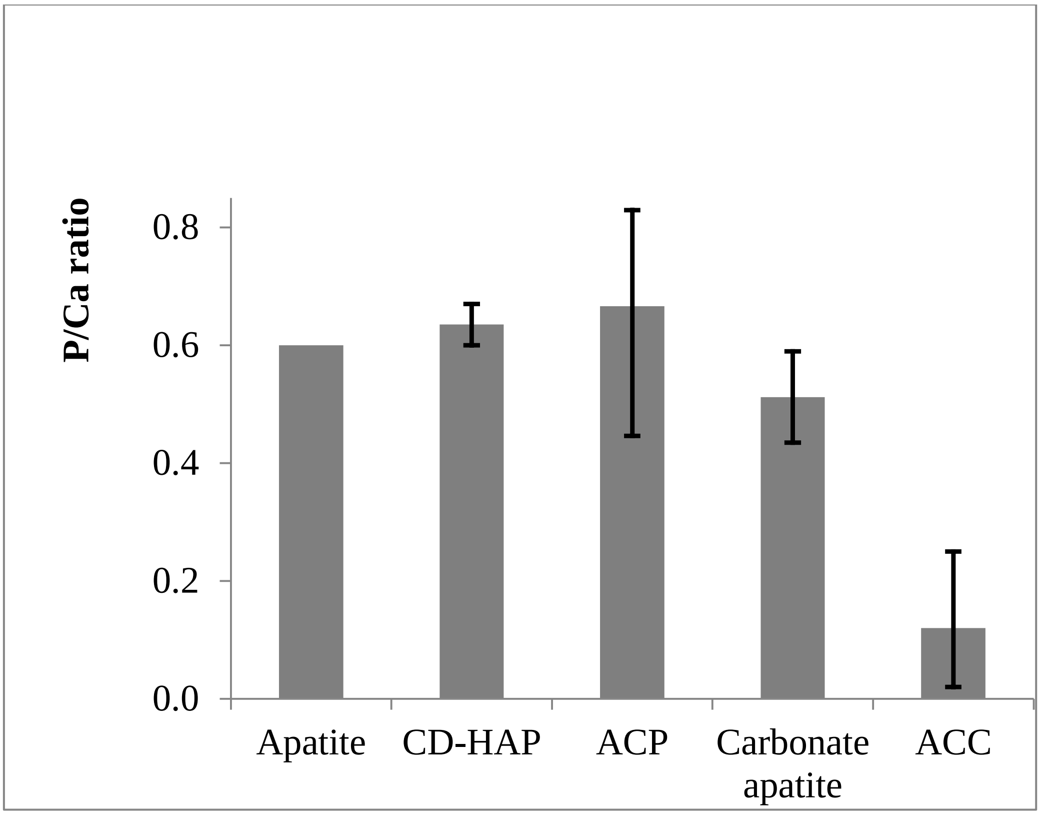

Figure S2. Typical P/Ca atomic ratios for apatite, calcium-deficient hydroxyapatite (CD-HAP) [1], carbonated apatite[2], ACP[3] and ACC[4]. The ratio of apatite (fluorapatite or hydroxyapatite) represents ideal stoichiometry of $Ca_{10}(PO_4)_6(OH, F)_2$. The carbonate- apatite that shows variability of P/Ca ratio is of type B (CO_3 substituting for PO_4) [2]. For ACP the literature provides many references with different P/Ca atomic ratio. The most frequent form of ACP has a ration P/Ca= 0.67, however, the ratio can be at the range of 0.45- 0.83 [3]. For ACC we use the P/Ca values (0.02-0.25) of biogenic ACC from the crayfish gastrolith[4]. In crustacean mandibles, the deviations from the typical values to lower ratios are probably due to carbonate substitution for phosphate which decreases the P/Ca ratio.

Supplementary Table S1

EPMA-WDS analyses of a molar tooth surface of *C. quadricarinatus* (Australian red claw crayfish) confirm a fluorapatite composition. Beam conditions: 15 keV and 15 nA. for detailed method description see Materials and Methods, units in atom %.										
Element / Analysis	Ca	Err%	P	Err%	F	Err%	C	Err%	O	Err%
1	16.74	1.65	11.31	2.58	5.89	1.71	10.11	3.24	56.40	5.64
2	18.46	2.70	12.77	1.14	2.69	0.32	11.11	3.71	54.36	2.47
3	17.90	1.57	11.23	1.78	3.95	0.63	10.04	4.85	56.87	4.03
4	16.15	1.57	12.71	2.74	4.78	0.14	10.74	3.14	55.33	4.00
5	18.22	1.77	10.77	1.53	3.03	0.31	10.20	6.54	57.72	3.15
6	16.37	1.42	11.61	2.82	2.37	1.64	13.82	8.84	55.81	3.29
Average	17.31		11.73		3.79		11.00		56.08	

Supplementary Table S2

EPMA-WDS analyses of a molar tooth surface of *S. mantis* (Mediterranean mantis shrimp) confirm a fluorapatite composition. Beam conditions: 15 keV and 15 nA. for detailed method description see Materials and Methods, units in atom %.										
Element / Analysis	Ca	Err%	P	Err%	F	Err%	C	Err%	O	Err%
1	17.74	0.38	10.77	0.74	3.18	0.44	11.18	2.63	57.88	1.49
2	17.74	0.38	10.90	0.74	3.15	0.13	11.56	3.30	57.29	1.41
3	17.18	0.37	11.23	0.72	3.68	0.32	10.55	7.74	57.33	1.39
4	17.75	0.37	12.08	0.72	3.21	1.51	10.52	6.49	57.08	1.39
5	17.17	0.38	11.92	0.72	2.98	2.40	10.59	5.88	57.80	1.42
Average	17.52		11.38		3.24		10.88		57.48	

Supplementary Video S1. Micro-CT volume rendered video of *S. mantis* mandible. The gray-scale images were pseudo-colored according to an RGB palette with low mineralization density in red and high density in blue. The incisor appears to be more robust than the molar ridge. Note the graded mineral density of each molar pointed cusp, with high density at the surface that gradually decreases towards the jaw. For scale bar see Fig. 5a.

1 Dorozhkin, S. V. & Epple, M. Biological and medical significance of calcium phosphates. *Angew. Chem. Int. Ed. Engl.* **41**, 3130-3146, doi:10.1002/1521-3773(20020902)41:17<3130::AID-ANIE3130>3.0.CO;2-1 (2002).

2 LeGeros, R. Formation and transformation of calcium phosphates: relevance to vascular calcification. *Z. Kardiol.* **90**, 116-124 (2001).

3 Dorozhkin, S. V. Amorphous calcium (ortho)phosphates. *Acta Biomater.* **6**, 4457-4475, doi:DOI 10.1016/j.actbio.2010.06.031 (2010).

4 Bentov, S., Weil, S., Glazer, L., Sagi, A. & Berman, A. Stabilization of amorphous calcium carbonate by phosphate rich organic matrix proteins and by single phosphoamino acids. *J. Struct. Biol.* **171**, 207-215, doi:DOI 10.1016/j.jsb.2010.04.007 (2010).
